# DNA-microarrays identification of *Streptococcus mutans *genes associated with biofilm thickness

**DOI:** 10.1186/1471-2180-8-236

**Published:** 2008-12-29

**Authors:** Moshe Shemesh, Avshalom Tam, Miriam Kott-Gutkowski, Mark Feldman, Doron Steinberg

**Affiliations:** 1 Biofilm Research Laboratory, Institute of Dental Sciences, Faculty of Dental Medicine, Hebrew University-Hadassah, POB 12272, Jerusalem 91120, Israel; 2 Microarray Service Laboratory, Core Research Facility at the Medical School, Hebrew University of Jerusalem, Israel

## Abstract

**Background:**

A biofilm is a complex community of microorganisms that develop on surfaces in diverse environments. The thickness of the biofilm plays a crucial role in the physiology of the immobilized bacteria. The most cariogenic bacteria, mutans streptococci, are common inhabitants of a dental biofilm community. In this study, DNA-microarray analysis was used to identify differentially expressed genes associated with the thickness of *S. mutans *biofilms.

**Results:**

Comparative transcriptome analyses indicated that expression of 29 genes was differentially altered in 400- vs. 100-microns depth and 39 genes in 200- vs. 100-microns biofilms. Only 10 *S. mutans *genes showed differential expression in both 400- vs. 100-microns and 200- vs. 100-microns biofilms. All of these genes were upregulated.

As sucrose is a predominant factor in oral biofilm development, its influence was evaluated on selected genes expression in the various depths of biofilms. The presence of sucrose did not noticeably change the regulation of these genes in 400- vs. 100-microns and/or 200- vs. 100-microns biofilms tested by real-time RT-PCR.

Furthermore, we analyzed the expression profile of selected biofilm thickness associated genes in the *luxS*^- ^mutant strain. The expression of those genes was not radically changed in the mutant strain compared to wild-type bacteria in planktonic condition. Only slight downregulation was recorded in SMU.2146c, SMU.574, SMU.609, and SMU.987 genes expression in *luxS*^- ^bacteria in biofilm vs. planktonic environments.

**Conclusion:**

These findings reveal genes associated with the thickness of biofilms of *S. mutans*. Expression of these genes is apparently not regulated directly by *luxS *and is not necessarily influenced by the presence of sucrose in the growth media.

## Background

In nature, most microorganisms possess the ability to attach to solid surfaces and to develop a densely packed community – a biofilm [1]. In the biofilm ecological niche, bacteria exhibit increased resistance to antimicrobial compounds, environmental stresses and host immune defense mechanisms [2,3]. The environmental heterogeneity that develops in biofilms can accelerate phenotypic and genotypic diversity in bacterial populations that allows them to accomodate adverse ecological conditions within the biofilm. Oral biofilms harboring pathogenic bacteria are among the major virulent factors associated with dental diseases such as caries, gingivitis and periodontal diseases [4,5]. Streptococci, including mutans streptococci, are ubiquitous in the oral microbiota of humans. *S. mutans *is considered to be the most important etiological agent in dental caries. It forms biofilms on tooth surfaces and causes dissolution of enamel by acid end-products resulting from carbohydrate metabolism [6-8].

The dental biofilm is the net result of a community of bacteria cooperating to form well-differentiated structures with distinct thickness [9,10]. Cells in the biofilm have unique phenotypic characteristics, which are different from their planktonic counterparts [1,11], accompanied by significant changes in their patterns of gene expression [12,13]. Our previous, in vitro comparative transcriptome analysis confirmed the hypothesis that there are significant changes in the pattern of gene expression following the transition of bacteria to biofilm growth modes [14]. In this study, DNA-microarrays and real-time RT-PCR analysis were carried out to characterize the transcriptional differences of this bacterium in various biofilm depths.

## Results and discussion

**Figure 1:**
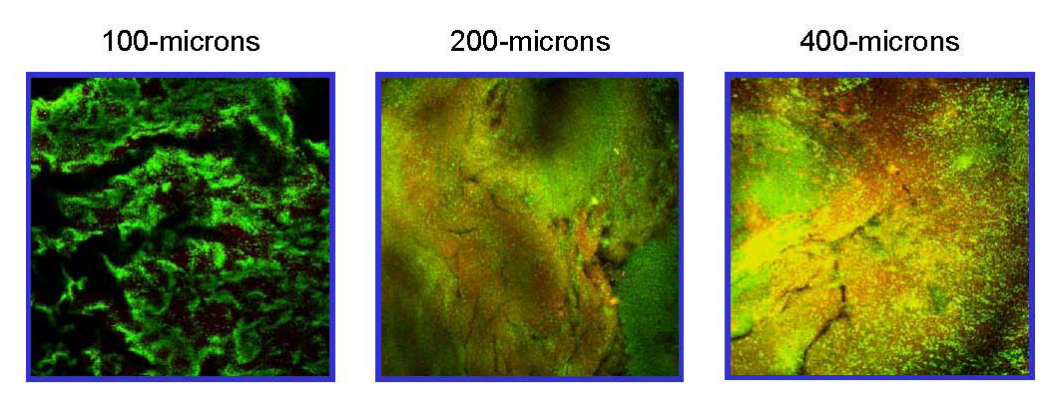
**Live/dead staining of biofilms grown at different depths**. *S. mutans *UA159 was grown at different biofilm depths in BHI, stained with LIVE/DEAD *Bac*Light fluorescent dye and analyzed with CLSM. The panels show images of 100-microns, 200-microns and 400-microns depths biofilms cultivated in the CDFF. Dead cells are stained red, and live cells are stained green. The images are representative of three biological experiments.

Viability and growth of bacteria within the biofilm is influenced by numerous environmental factors, such as nutrition supply, outflux of metabolites, pH gradient and oxygen tension [1,9,15]. Lack of nutrition and accumulation of toxic products may account for a decline in the growth of bacteria in suspension and in biofilm. One of the major rate-controlling factors in a biofilm ecosystem is the diffusion rate of nutrients in and waste products out and gases across the biofilm [9,15]. A thinner, or a low-density biofilm with void volumes and water channels may facilitate easier transport of nutrients in and waste products out across the biofilm, thus having a limited effect on bacterial proliferation [16,17]. It is conceivable therefore that the gene expression profile in thicker biofilms may be attributed to the effects of nutrient limitation to a large portion of the biomass due to diffusion restrictions. As biofilm thickness plays a paramount role, we conducted this study under controlled nutrient flow and controlled biofilm depth of 100, 200 and 400 microns, by using the constant-depth film fermenter (CDFF). The biofilms cultivated in the CDFF were assessed by confocal laser scanning microscopy (CLSM). The CLSM images demonstrated that biofilm maturation and increased thickness is accompanied by significant alterations in cell viability in the different biofilm layers. According to our results, comparative vitality of bacteria grown at 100-micron depth was much greater than those cultured in CDFF at 200- or 400-microns depths (Fig. 1). This decrease in viability might be due to restriction in nutrient availability and accumulation of toxic metabolites as the biofilm thickens. Therefore it is reasonable that differential gene expression will allow the bacteria to acclimate in different ecological microenvironments.

Recent studies have revealed that the regulated death of bacterial cells is important for biofilm development. Following cell death, a subpopulation of the dead bacteria lyses and releases genomic DNA, which then has a central role in intercellular adhesion and biofilm stability [18]. Cell fate is generally thought of as being deterministic. That is, the fate that cells adopt is in most cases governed by the history of the cell or its proximity to inductive signals from other cells. However, an increasing number of cases are now known in which cell fate is controlled by stochastic (deterministic) mechanisms, for instance entry into the persister state in *E. coli *[19,20]. Stochastic fluctuations in the cellular components that determine cellular states can cause two distinct subpopulations, a property called bistability. Bistable switches produce polarized expression states and result in subpopulations of specialized cell types that are either ON or OFF for gene expression. Polarized output is thought to arise when a transcriptional regulator reaches a threshold activity and initiates an auto-regulatory positive feedback loop [21,22]. Below the threshold, the regulator does not auto-activate and the system remains in the OFF state. Above the threshold, runaway positive feedback results in activation of the system and stabilization of the ON state. Several properties in *B. subtilis*, the phenotypes of motility, genetic competence, sporulation and biofilm formation, are regulated by bistable gene expression [22-24]. It has been also demonstrated [24] that the genes encoding the components of the extracellular matrix in *B. subtilis *are expressed only in a subpopulation of cells. Matrix production is energetically costly and choosing the subset of cells within a given population to be responsible for producing and providing the extracellular polysaccharides (EPS) for the entire biofilm community might be especially economical. In the context of our results, the heterogeneity in the viable population of bacteria within the deep layers of a mature biofilm might be a result of a strategy to relegate the energetic cost to a subpopulation that would provide strengthening for the entire community. Phenotypic diversity within bacterial populations is advantageous. Multiple cell types permit a "division of labor", and cells with specialized properties may be primed to exploit or resist changes in the environment [21]. Consequently, biofilms should contain specialized cell types that are differentiated by transcriptional regulation, to express different phenotypic characteristics.

**Figure 2:**
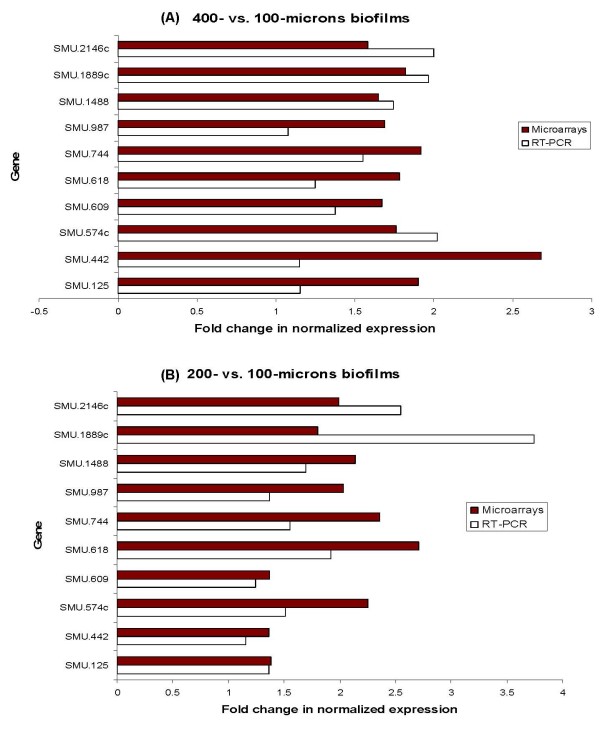
**Comparison of microarrays and RT-PCR expression data**. Comparison of microarrays and RT-PCR expression values for selected genes of *S. mutans*, grown in different biofilm depths in CDFF. The data are expressed as the means of at least two biologically independent experiments.

DNA-microarray analysis was used to identify differentially expressed genes associated with the thickness of *S. mutans *biofilm at various depths. Analyses of microarrays images, using an empirical Bayesian method (B-test) [25], indicated that expression of 29 genes was differentially changed in 400- vs. 100-microns depth biofilms (Table 1), and 39 genes in 200- vs. 100-microns depth biofilms (Table 2), at a confidence level of *P *≤ 0.05 tested by moderated *t*-test. About 11% of these genes code for membrane-related proteins, while almost one-half of the differentially regulated genes code for hypothetical proteins of as yet unknown function present in the *S. mutans *genome. Only 10 genes of *S. mutans *(Table 3) showed differential expression in both 400- vs. 100-microns and 200- vs. 100-microns biofilms. All of these genes were upregulated. Verification of the microarrays data was carried out using real-time RT-PCR for expression analysis of selected differentially regulated genes (Fig. 2). A substantial number of them, such as SMU.574c, SMU.609, and SMU987, appeared to code for cell wall-associated proteins. SMU.987 encodes a cell wall-associated protein precursor WapA, a major surface protein [26], which modulates adherence and biofilm formation in *S. mutans*. Previous studies have demonstrated that levels of *wapA *in *S. mutans *were significantly increased in biofilm phase [27], whereas the inactivation of *wapA *resulted in reduction in cell aggregation and adhesion to smooth surfaces [28]. The *wapA *mutants are of reduced cell chain length, have less sticky cell surface, and unstructured biofilm architecture compared to the wild-type [29].

**Table 1:** The most significant (*P *≤ 0.05) differentially expressed genes of *S. mutans *in 400 μm vs. 100 μm biofilms

Locus number^*a*^	Description^*a*^	M^*b*^	*P *value^*c*^	B^*d*^
SMU.173	putative ppGpp-regulated growth inhibitor	1.021	0.001	5.781
SMU.1710c	conserved hypothetical protein	1.246	0.001	5.520
SMU.991	putative ribonucleotide reductase	0.932	0.004	4.187
SMU.1080c	conserved hypothetical protein possible transposon-related protein	-0.635	0.013	2.948
SMU.564	conserved hypothetical protein	0.666	0.018	2.409
SMU.1974	putative pyrroline carboxylate reductase	0.696	0.018	2.267
SMU.1758c	conserved hypothetical protein	-0.666	0.028	1.676
SMU.125	conserved hypothetical protein	0.914	0.028	1.564
SMU.359	translation elongation factor G (EF-G)	0.978	0.028	1.477
SMU.59	adenylosuccinate lyase	0.691	0.028	1.381
SMU.987	cell wall-associated protein precursor WapA	0.760	0.028	1.313
SMU.85	putative phosphomethylpyrimidine kinase	0.623	0.032	1.101
SMU.2146c	hypothetical protein	0.656	0.036	0.963
SMU.37	phosphoribosylaminoimidazolecarboxamide formyltransferase/IMP cyclohydrolase	0.540	0.036	0.786
SMU.02	putative DNA polymerase III, beta subunit	0.605	0.036	0.775
SMU.609	putative 40K cell wall protein precursor	0.739	0.04	0.575
SMU.574c	putative membrane protein	0.814	0.04	0.487
SMU.1353	putative transposase	0.461	0.04	0.485
SMU.1839	mannose-6-phosphate isomerase	0.590	0.041	0.471
SMU.641	putative oxidoreductase	-0.595	0.044	0.225
SMU.1889c	hypothetical protein	0.860	0.044	0.213
SMU.442	conserved hypothetical protein	1.416	0.044	0.171
SMU.1646c	conserved hypothetical protein, possible hemolysis inducing protein	1.031	0.044	0.157
SMU.229	conserved hypothetical protein	-0.480	0.045	0.096
SMU.1760c	conserved hypothetical protein	-0.548	0.047	0.016
SMU.84	putative tRNA pseudouridine synthase A	0.715	0.048	-0.056
SMU.2005	putative adenylate kinase	-0.577	0.05	-0.147
SMU.1488c	conserved hypothetical protein	0.725	0.05	0.033
SMU.1642c	conserved hypothetical protein	0.771	0.05	-0.196

^*a *^Based on the genome annotation of *S. mutans *provided by TIGR.

^*b *^Log_2_-fold expression according to the M value, M > 0 means upregulation and M < 0 downregulation of the gene.

^*c *^Moderated *t*-test and its corresponding *P *value, adjusted by Benjamini and Yekutiely method.

^*d *^Bayesian test value, meaning the probability for a gene to be differentially expressed. Genes are listed according to their decreasing statistical importance according to their B-value.

**Table 2:** The most significant (*P *< 0.05) differentially expressed genes of *S. mutans *in 200 μm vs. 100 μm biofilms

Locus number^*a*^	Description^*a*^	M^*b*^	*P *value^*c*^	B^*d*^
SMU.574c	putative membrane protein	1.169	0.006	4.950
SMU.1782	conserved hypothetical protein	1.250	0.016	2.504
SMU.744	putative cell division protein FtsY signal recognition	1.236	0.016	2.545
SMU.1889c	hypothetical protein	0.850	0.016	2.536
SMU.1537	putative glycogen biosynthesis protein GlgD	0.598	0.016	2.395
SMU.1488c	conserved hypothetical protein	1.103	0.018	2.187
SMU.402	pyruvate formate-lyase	0.788	0.019	1.814
SMU.943c	putative hydroxymethylglutaryl-CoA synthase	-0.617	0.019	1.780
SMU.495	glycerol dehydrogenase	0.572	0.020	1.596
SMU.804	hypothetical protein	0.940	0.025	1.234
SMU.1578	putative biotin operon repressor	0.532	0.025	0.993
SMU.814	putative MutT-like protein	0.598	0.025	0.984
SMU.618	hypothetical protein	1.442	0.025	0.912
SMU.987	cell wall-associated protein precursor WapA	1.020	0.025	0.881
SMU.417	conserved hypothetical protein	-0.622	0.025	0.866
SMU.929c	conserved hypothetical protein	-0.562	0.028	0.776
SMU.125	conserved hypothetical protein	0.457	0.032	0.518
SMU.1841	putative PTS system, sucrose-specific IIABC component	0.782	0.038	0.300
SMU.609	putative 40K cell wall protein precursor	0.454	0.042	0.144
SMU.1626	50S ribosomal protein L1	0.897	0.042	0.401
SMU.1298	50S ribosomal protein L31	-0.692	0.042	0.081
SMU.630	hypothetical protein	-0.483	0.042	0.036
SMU.892	putative type I restriction-modification system, specificity determinant	-0.745	0.043	0.067
SMU.2105	hypothetical protein	0.862	0.043	-0.127
SMU.2146c	hypothetical protein	0.993	0.043	-0.146
SMU.1975c	conserved hypothetical protein possible membrane protein	0.900	0.043	0.182
SMU.102	putative PTS system, IID component	-0.422	0.045	-0.218
SMU.149	putative transposase	0.795	0.046	-0.285
SMU.883	dextran glucosidase DexB	0.393	0.046	-0.322
SMU.99	fructose-1,6-biphosphate aldolase	0.510	0.046	-0.331
SMU.27	putative acyl carrier protein AcpP	0.809	0.047	-0.059
SMU.91	peptidyl-prolyl isomerase RopA (trigger factor)	0.544	0.049	-0.525
SMU.1421	putative dihydrolipoamide acetyltransferase, E2 component	0.565	0.049	-0.564
SMU.886	galactokinase, GalK	0.676	0.049	-0.577
SMU.753	conserved hypothetical protein	-0.700	0.049	-0.592
SMU.442	conserved hypothetical protein	0.435	0.049	-0.600
SMU.1854	conserved hypothetical protein	-0.748	0.049	-0.229
SMU.940c	putative hemolysin III	-0.651	0.049	-0.473

^*a *^Based on the genome annotation of *S. mutans *provided by TIGR.

^*b *^Log_2_-fold expression according to the M value, M > 0 means upregulation and M < 0 downregulation of the gene.

^*c *^Moderated *t*-test and its corresponding *P *value, adjusted by Benjamini and Yekutiely method.

^*d *^Bayesian test value, meaning the probability for a gene to be differentially expressed. Genes are listed according to their decreasing statistical importance according to their B-value.

**Table 3:** Nucleotide sequences of primers for genes whose expression in both 400 μm vs. 100 μm and 200 μm vs. 100 μm biofilms was compared

ORF^*a*^	Primer Sequences (5' – 3')	
	
	Forward	Reverse
16S rRNA	CCTACGGGAGGCAGCAGTAG	CAACAGAGCTTTACGATCCGAAA
SMU.125	TGGCACATGCACGAGAAGAA	GGCCCGGAATAGCATAGTTG
SMU.442	CTTTCAGGCGGGATGTTGAG	GCTGTCTGGCGGTTTCAATC
SMU.574c	TGGTCATACAGTTGTGCAGC	GACGAGGCCGATGCAACA
SMU.609	CAGTTGTGAACGTGGCTGAAA	TGAGCTGCTGCCTTATCTGAAA
SMU.618	GCACTTATCGCTTGCGGTTT	CACCTGACAATACCAGCAACCA
SMU.744	TGTTCAGGTTGCGTCAACCTT	AATGACACGGCGAAGAGCTT
SMU.987	GCACGCTTGCAGTACATTGC	CATAAGGTCGCGAGCAGCT
SMU.1488c	ACGCCATCTCATCAGCCTTT	ACCACTTACCCAATCGCTTGA
SMU.1889c	CTGCTGTATCTGCCCCTGCT	CCTAAATCCCAACCAATTGCTG
SMU.2146c	CAGGAGCTTCAGGTCTCTTTCAAA	CCTTGGGCTTTATAAGCATTGATAG

^*a *^Based on the genome annotation of *S. mutans *provided by TIGR

SMU.744, encoding membrane-associated receptor protein FtsY, the third universally conserved element of the signal recognition particle (SRP) translocation pathway [30], was also found among these differentially expressed genes. SRP was first identified in mammalian cells, and later in bacteria, and it was further shown that components of the SRP pathway are universally conserved in all three domains of life – archaea, bacteria, and eukaryote domains [31]. The SRP pathway delivers membrane and secretory proteins to the cytoplasmic membrane or endoplasmic reticulum [32]. *S. mutans *remained viable but physiologically impaired and sensitive to environmental stress when *ftsY *and other genes of the SRP elements were inactivated [30]. The upregulation of FtsY in the deep layers of the biofilm indicates that the SRP system is crucial for bacterial survival in highly condensed mature biofilm.

Interestingly, relatively few of the differentially expressed genes showed more than a 2-fold change. However, even small changes in mRNA levels could have the biological potential to affect bacterial metabolism and physiology. Relatively small changes in the level of expression of one gene can be amplified through regulatory networks and result in significant phenotypic alteration [33]. Many genes among those upregulated in 200- vs. 100-microns biofilms are involved in energy metabolism, including SMU.99, SMU.402, SMU.883, SMU.886 and SMU.1537. However, a substantial number – 11% of the upregulated genes – code for cell wall-associated proteins, such as SMU.574c and SMU.609. Several differentially expressed genes in the 400- vs. 100-microns depths are presumably regulatory genes, e.g. SMU.173 and SMU.359, which influence biofilm formation.

**Figure 3:**
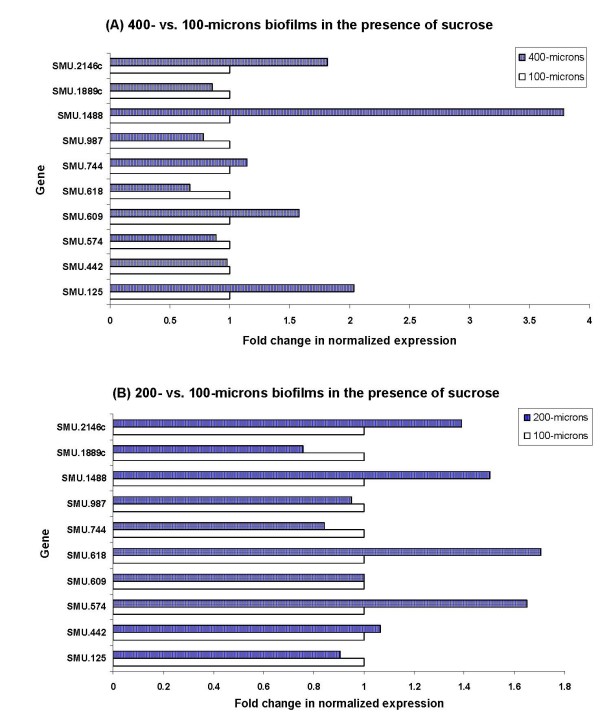
**Gene expression of *S. mutans *in the presence of sucrose**. Gene expression of *S. mutans *grown at various biofilm depths in the presence of 2% sucrose. The data are expressed as the means of at least two biologically independent experiments.

Previous studies have shown that carbohydrates have a major influence on biofilm-associated gene expression of several types of bacteria [34-37]. Sucrose-dependent adhesion is a major mechanism of biofilm formation and maturation in dental biofilms [34-37]. In order to evaluate whether sucrose influences the expression of selected genes of *S. mutans*, we generated biofilms of 100-, 200- and 400-micron depths in the presence of 2% sucrose. It is of interest that the presence of sucrose did not dramatically change the regulation of the selected genes in 400- vs. 100-microns (Fig. 3A) or 200- vs. 100-microns biofilms (Fig. 3B) tested by real-time RT-PCR. Only SMU.1488 of the tested genes showed significant upregulation in 400- vs. 100-microns depth of biofilm with sucrose (Fig. 3A). A possible explanation of these results could be that sucrose affects mostly the initial stages of biofilm formation, as generation of the sticky glucans and fructans is especially crucial in the initial stages of adhesion [6,34], while the biofilm thickness-associated genes are activated mostly in the late steps of biofilm development. The genes, which appear to be responsible for biofilm thickness and maturation processes, are not necessarily influenced by the presence of sucrose during the initial biofilm formation stage.

**Figure 4:**
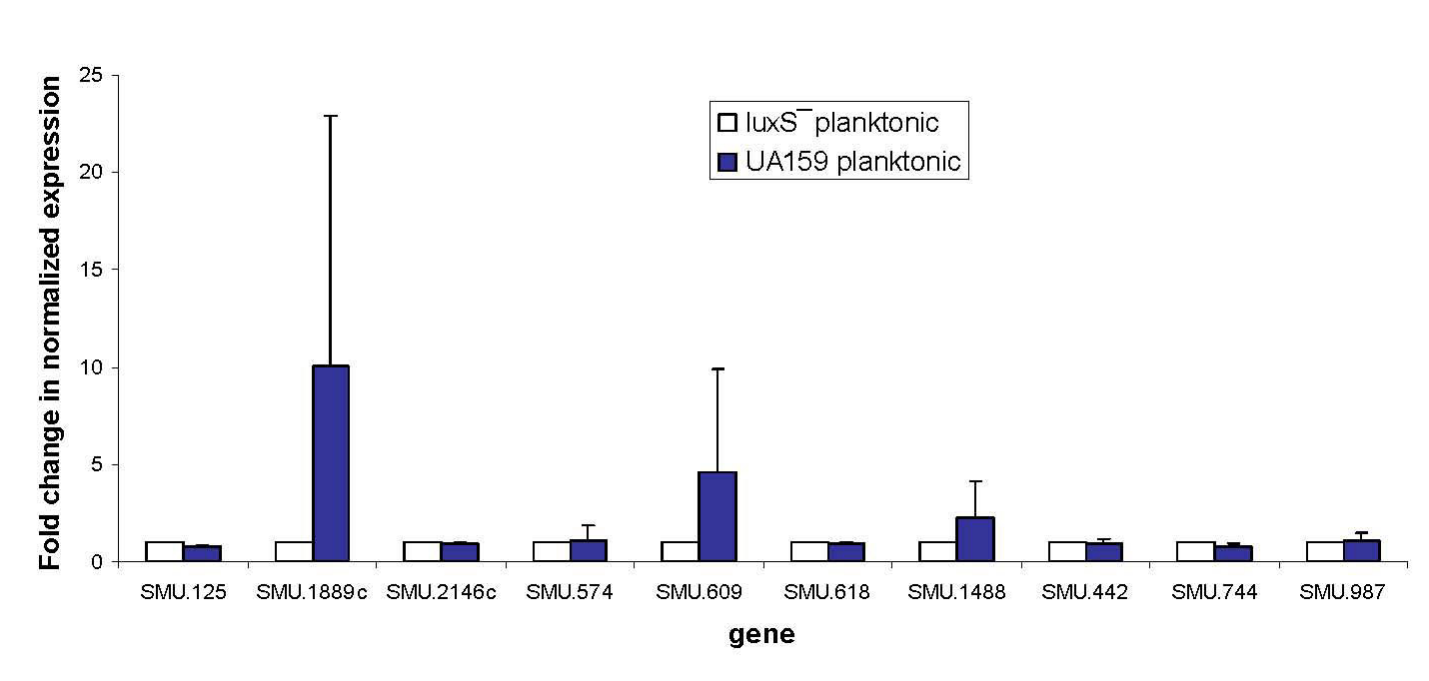
**Regulation of *S. mutans *genes expression**. Regulation of selected gene expression of *S. mutans *UA159 compared with the *luxS*^- ^strain in planktonic growth examined by real-time RT-PCR. The data are expressed as the means and standard deviations of at least two biologically independent experiments.

**Figure 5:**
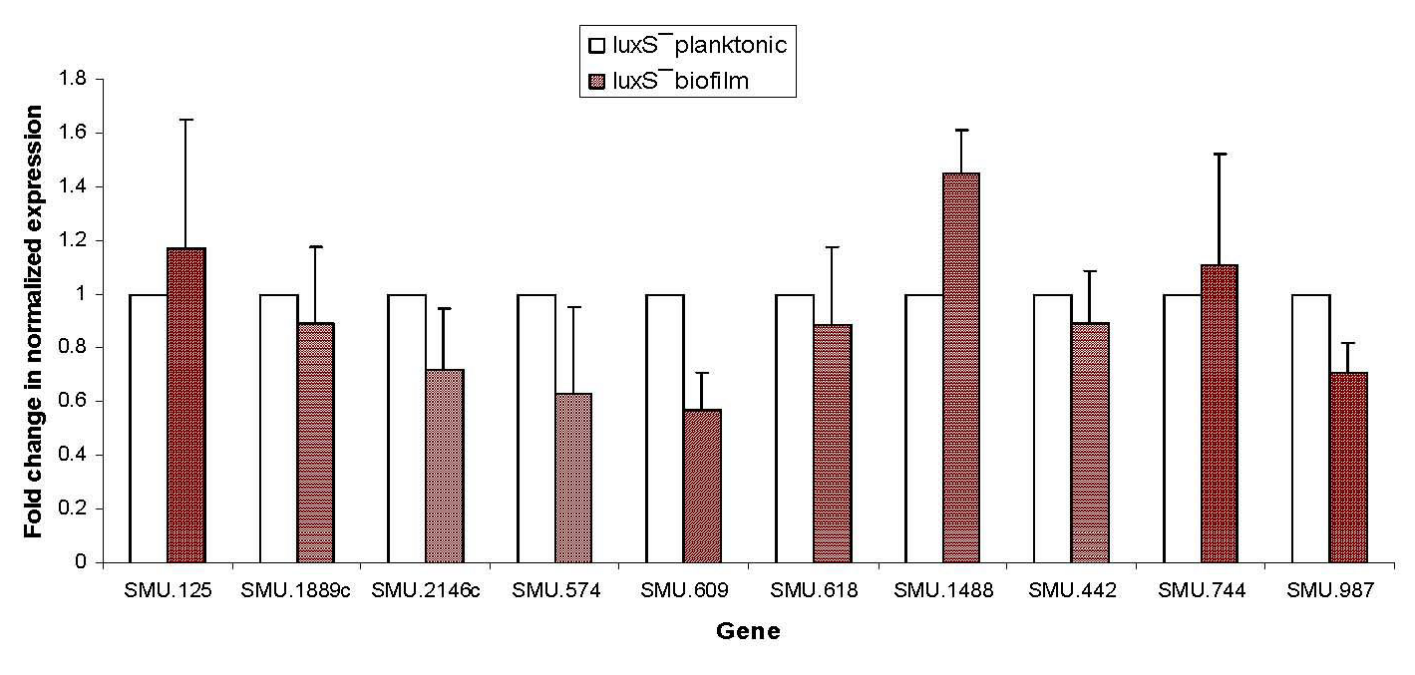
**Regulation of *S. mutans *gene expression**. Regulation of selected gene expression of *S. mutans luxS*^- ^examined by real-time RT-PCR in biofilm compared with planktonic cultures. The data are expressed as the means and standard deviations of at least two biologically independent experiments.

Cell-cell communication plays an important role in the successful formation, survival and virulence of the biofilm community [38-41]. Gram-positive bacteria generally communicate via small diffusible peptides [40,42], while many Gram-negative bacteria secrete acyl homoserine lactones (AHLs) [42,43], the structure of which varies depending on the species of bacteria that produce them. Another system associated with quorum sensing (QS) involves the synthesis of autoinducer-2 (AI-2), which is derived from a common precursor, 4,5-dihydroxy-2,3-pentadione (DPD), the product of the LuxS enzyme [44,45]. This system may be involved in cross-communication among both Gram-positive and Gram-negative bacteria, as homologues of LuxS are widespread within the microbial world. LuxS is highly conserved in the bacterial kingdom [46-50]. In *S. mutans*, QS regulates cardinal physiological functions, such as the ability to withstand environmental stress conditions, competence and biofilm formation [51,52]. Knockout of the *luxS *gene was shown to impair biofilm growth and stress tolerance of bacteria [46,52,53]. As a step towards understanding the possible link between biofilm thickness and QS, we analyzed the profile of selected gene expression in a *luxS*^- ^mutant strain. The expression of genes identified in this study as associated with biofilm thickness was first compared between the mutant and wild-type strains in planktonic condition, and afterwards under biofilm vs. planktonic environments in the *luxS*^- ^strain. Interestingly, there were no radical changes in expression of genes associated with biofilm thickness in the tested conditions (Fig. 4). Only minor downregulation was recorded in SMU.2146c, SMU.574, SMU.609, and SMU.987 genes expression in biofilm vs. planktonic conditions (Fig. 5). This result may explain the observation that biofilms of *S. mutans *deficient in *luxS *were not radically different from the wild-type [54]. Although *luxS *plays an important role in initial adherence and initiation of mature biofilm development, it seems that biofilm thickness-associated genes are not regulated directly by *luxS *in *S. mutans *UA159. Interestingly, the *luxS*^- ^mutant showed a diminished capacity to form biofilm when sucrose was provided as a supplemental sugar [52], so it can be suggested that *luxS *plays a significant role, most likely in regulation of sucrose-dependent adherence rather than cell-cell interactions during biofilm growth in *S. mutans*.

## Conclusion

This study provides a genome-scale outline of genes associated with biofilm thickness in *S. mutans*, a highly pathogenic bacterium in dental diseases. By expression alterations in these genes, the bacteria within the various layers of a mature biofilm may express different phenotypic characteristics allowing better acclimation in the biofilm micro-environment. Moreover, the expression of these genes is not directly regulated by *luxS *and is not necessarily influenced by the presence of sucrose during biofilm maturation.

## Methods

### Bacterial strains and culture conditions

*S. mutans *UA159 and its derivative mutant strain *luxS*^- ^[54] were incubated in Brain Heart Infusion Broth (BHI, Difco Labs, Detroit, USA) at 37°C in 95% air/5% CO_2 _(v/v), with the addition of erythromycin (10 μg/ml) in the case of the *luxS*^- ^strain. Cultures of *S. mutans *were diluted 1:50, inoculated into fresh BHI media and grown in polystyrene tubes for 24 h (37°C, 95% air/5% CO_2 _(v/v)) for planktonic culture generation. The biofilm of *luxS*^- ^was grown in BHI with addition of erythromycin (10 μg/ml) in 20-mm diameter, 15-mm deep sterile polystyrene multidishes (NUNCLON-143982, Roskilde, Denmark), as described previously [14].

As biofilm thickness plays a crucial role in mature biofilm development, we generated biofilms of wild-type bacteria under controlled nutrition flow and controlled biofilm depth conditions, by using the constant depth film fermentor (CDFF) [55]. The rotating turntable in the CDFF contained 15 polytetrafluoroethylene (PTFE) pans, rotated under PTFE scraper bars that smear the incoming medium over the 15 pans. Each sampling pan contained 5 cylindrical holes (5.0 mm in diameter) with PTFE plugs on which biofilms were formed. The desired depth of biofilm was achieved using a recessing tool, which pushes the plug down to the required depth (100, 200 or 400 μm).

The biofilms were grown for 72 h at different biofilm depths of 100, 200 and 400 microns, as follows: pure cultures of *S. mutans *UA159 were cultivated overnight at 37°C in 700 ml of BHI. Next, the inoculum was pumped into the CDFF for 7 h via a peristaltic pump (ISMATEC SA, Labortechnik-Analytik, Zurich, Switzerland) at a rate of 100 ml/h at 37°C. BHI was delivered into the CDFF at a rate of 100 ml/h. After 72 h at 37°C, the PTFE cylindrical plugs, on which the biofilms of different depths were grown, were carefully removed from the CDFF. The collected biofilms were washed gently with sterile PBS, and the biofilm bacteria were harvested and subjected to RNA extraction. We also tested whether the addition of sucrose to the growth medium had any influence on the expression of the selected genes (Table 3) of *S. mutans *biofilms grown to 100-, 200- and 400-microns depths; the biofilm samples were generated and grown for 72 h in CDFF with unsupplemented BHI, and BHI supplemented with 2% sucrose (Frutarom Ltd, Israel).

### Confocal laser scanning microscopy

The biofilm samples developed on plugs which were not used for the gene expression analysis were stained with LIVE/DEAD *Bac*Light fluorescent dye (Molecular Probes, OR) (1:100) for 10 min. Dead bacteria are stained red while the live ones are stained green. Fluorescent images of the PBS washed samples were assessed using a Zeiss LSM 410 (Carl Zeiss Microscopy, Jena, Germany) confocal laser scanning microscope (CLSM), equipped with PlonNeofluor × 10 lens (Zeiss). In each experiment, exciting laser intensity, background level, contrast and electronic zoom size were maintained at the same level. At least three random fields were analyzed in each experiment. A series of optical cross-sectional images were acquired from the surface through the vertical axis of the specimen, using a computer-controlled motor drive. 3-D confocal images were reconstituted with Image Pro Plus 4.2 (Media Cybernetics, Silver Spring, MD) and processed for display using Adobe Photoshop Ver 7.0 software. The thickness of the biofilm was controlled and determined by the CDFF apparatus; estimation of live/dead ratio was assessed according to the CLSM. Due to limitations of the CLSM technique, the 400-microns depth biofilm calculation does not necessarily include the entire biofilm depth [56].

### RNA extraction

Extraction of total RNA from bacteria grown in biofilms as well as planktonically grown cells was performed as described previously [14]. The RNA concentration was determined spectrophotometrically using the Nanodrop Instrument (ND-1000, Nanodrop Technologies, Wilmington, DE, USA). The integrity of the RNA was examined by agarose-gel electrophoresis (data not shown).

### Microarrays design, cDNA labeling and hybridization

The arrays consisted of 1948 70-mer oligonucleotides representing 1960 open reading frames (ORF) from *S. mutans *UA159 and additional control sequences. The probe labeling, hybridization and array data normalization were carried out as previously described [14]. In brief, cDNA was generated with random primers from total RNA and labeled indirectly with cy3 or cy5 dyes. The hybridizations were all performed against the 100-micron biofilm in a reference design manner. The slides were scanned using a Genepix 4000B scanner (Axon Ltd). Fluorescence intensities were quantitatively analyzed using GenePix Pro 4.1 software (Axon). The result files (gpr) produced by GenePix were analyzed utilizing the LIMMA software package [57], available from the CRAN site . After filtering, the data within the same slide were normalized using global loess normalization with a default smoothing span of 0.3 [58]. To identify differentially expressed genes, a parametric empirical Bayes approach implemented in LIMMA was used [25]. According to this approach, data from all the genes in a replicate set of experiments are combined into estimates of parameters of a priori distribution. These parameter estimates are then combined at the gene level with means and standard deviations to form a statistic B that is a Bayes log posterior odds [25]. B can then be used to determine whether differential expression has occurred. A moderated *t*-test was performed in parallel, with the use of a false discovery rate correction for multiple testing [59]. TIGR arrays include four replicates for each gene. Instead of just taking the average of replicate spots, we used the duplicate correlation function [60] available in LIMMA to acquire an approximation of gene-by-gene variance. This method greatly improves the precision with which the gene-wise variances are estimated and thereby maximizes inference methods designed to identify differentially expressed genes. A *P *value < 0.05 confidence level was used to pinpoint the significantly differentiated genes. Genes had to have an *A*-value (A = log_2 _[Cy3 × Cy5]/2), the average expression level for the gene across all arrays and channels) of more than 8.5, leaving out genes with an average intensity in both channels less than 256. The microarray data were deposited in the GEO public repositories with accession number GSE12496.

### Reverse transcription and real-time quantitative PCR

Quantitative SYBR green PCR assays employing an ABI-Prism 7300 Light Cycler System (Applied Biosystems, Foster City, CA, USA) were performed as described previously [36]. The corresponding oligonucleotide primers were designed using the algorithms provided by Primer Express (Applied Biosystems) for uniformity in size (≈ 90 base-pairs) and melting temperature. For each set of primers, a standard amplification curve was plotted (critical threshold cycle against log of concentration), and only those with slope ≈ -3 were considered reliable primers. The expression levels of all the tested genes for real-time RT-PCR were normalized using the 16S rRNA gene of *S. mutans *(Acc. No. X58303) as an internal standard. Each assay was performed with at least two independent RNA samples in duplicate.

## Abbreviations

AHL: acyl homoserine lactone; AI-2: autoinducer-2; BHI: brain heart infusion; CDFF: constant-depth film fermenter; CLSM: confocal laser scanning microscope; EPS: extracellular polysaccharides; QS: quorum sensing; PTFE: polytetrafluoroethylene; RT-PCR: reverse transcription polymerase chain reaction; TIGR: The Institute for Genomic Research.

## Authors' contributions

MS planed and carried out the experiments, performed the array and real time RT-PCR analyses and wrote the original manuscript. AT assisted in biofilms generation, RNA extraction, RT-PCR and CLSM experiments. MK performed the probe labeling, hybridization and array data normalization for DNA-microarrays. MF helped in setting up and performing the CDFF experiments to generate different depths of biofilm. DS conceived the study and oversaw its execution; he also revised the manuscript critically for important intellectual content. MS and DS integrated all of the data throughout the study and crafted the final manuscript. All authors read and approved the manuscript.
